# A Study on Structural Health Monitoring of a Large Space Antenna via Distributed Sensors and Deep Learning

**DOI:** 10.3390/s23010368

**Published:** 2022-12-29

**Authors:** Federica Angeletti, Paolo Iannelli, Paolo Gasbarri, Massimo Panella, Antonello Rosato

**Affiliations:** 1 School of Aerospace Engineering, Sapienza University of Rome, Via Salaria 851, 00138 Rome, Italy; 2 Since February 1st 2022—OHB System AG, Manfred-Fuchs-Straße 1, 82234 Weßling, Germany; 3 Department of Mechanical and Aerospace Engineering (DIMA), Sapienza University of Rome, Via Eudossiana 18, 00184 Rome, Italy; 4 Department of Information Engineering, Electronics and Telecommunications (DIET), Sapienza University of Rome, Via Eudossiana 18, 00184 Rome, Italy

**Keywords:** space structures, space antenna, damage classification, deep learning, structural health monitoring

## Abstract

Most modern Earth and Universe observation spacecraft are now equipped with large lightweight and flexible structures, such as antennas, telescopes, and extendable elements. The trend of hosting more complex and bigger appendages, essential for high-precision scientific applications, made orbiting satellites more susceptible to performance loss or degradation due to structural damages. In this scenario, Structural Health Monitoring strategies can be used to evaluate the health status of satellite substructures. However, in particular when analysing large appendages, traditional approaches may not be sufficient to identify local damages, as they will generally induce less observable changes in the system dynamics yet cause a relevant loss of payload data and information. This paper proposes a deep neural network to detect failures and investigate sensor sensitivity to damage classification for an orbiting satellite hosting a distributed network of accelerometers on a large mesh reflector antenna. The sensors-acquired time series are generated by using a fully coupled 3D simulator of the in-orbit attitude behaviour of a flexible satellite, whose appendages are modelled by using finite element techniques. The machine learning architecture is then trained and tested by using the sensors’ responses gathered in a composite scenario, including not only the complete failure of a structural element (structural break) but also an intermediate level of structural damage. The proposed deep learning framework and sensors configuration proved to accurately detect failures in the most critical area or the structure while opening new investigation possibilities regarding geometrical properties and sensor distribution.

## 1. Introduction

Modern large space structures (LSS) have a higher level of structural flexibility and larger dimensions than those experienced in past spacecraft. Due to the constraints imposed by the launch vehicle on the lift-off mass, the size of such flexible appendages (i.e., antennas, telescopes, solar panels, etc.) comes at the expense of the overall stiffness of their structural elements. Therefore, many large structures exhibit a multitude of closely spaced and lightly damped elastic modes, thus making the Control/Structure Interaction (CSI) phenomenon a very critical issue that can significantly affect the satellite’s behaviour and degrade its performance. In fact, external and internal disturbances such as thermal loads, reaction wheels/mechanisms exported forces and vibrations, among others, or the control inputs for attitude stabilisation and orbital manoeuvres, can easily excite the vibrational responses of the satellites’ flexible appendages [1]. Moreover, flexible appendages are generally subjected to narrow pointing and instrument requirements [2,3,4]. However, during their operative life, such parts are potentially exposed to several sources of damage and degradation, as well as sudden stiffness loss, due to debris impact or fatigue and material ageing or wear phenomena. When a failure or deterioration occurs, a change in the overall system properties takes place. Even minor local structural damages may induce a relevant change in the stiffness and dimensional stability of spacecraft scientific instruments, possibly compromising the acquisition of data and images.

Spacecrafts such as the Soil Moisture Active Passive (SMAP) [5,6], launched by NASA in 2015, or the European Copernicus Imaging Microwave Radiometer (CIMR) [7], currently under development by Thales Alenia Space, can be mentioned as representative cases of such large flexible systems. Both the cited missions consist of a main non-spun platform and a rotating part composed of an antenna boom, a deployable large mesh reflector model (LMRM), and a rotation mechanism. The interest in such larger and structurally complex missions imposes the precise analysis of how, where, and when failures can be detected with sufficient accuracy. To this end, we analyse in this paper a satellite hosting an LMRM payload with properties and characteristics comparable to the SMAP and CIMR missions. In particular, the LMRM is deemed a case of interest given its design maturity, promising current and future space applications, and also its high-precision pointing and balance requirements [8], which make it particularly susceptible to performance degradation in case of local structural damages. The goal is to study the sensitivity of a distributed network of sensors to structural damages when using a deep learning approach.

On account of this, the current and next LSS and related missions are calling for new de-risking technologies, aimed at minimising the impact of damages directly on orbiting satellites. A possible approach is to implement a set of sensors—whose amount and location will depend on the specific application—to promptly identify the location and the entity of the damage, and to assess the impact on the mission and relevant mitigation strategies [9]. In such cases, Structural Health Monitoring (SHM) strategies have hence assumed an increasing importance on the consideration of improved performance, safety, and costs, especially referring to modern space lightweight structures [10,11]. An important aspect of the SHM of the LSS is related to the use of in situ sensing to detect the structural damage of the structures and their related components [12,13,14]. Different sensing technologies can be used to identify the damages of on-board satellites: the solutions range from cameras allowing image processing (such as the debris hit detection on Sentinel 1-A’s solar panel) to distributed devices—such as accelerometers, piezoelectrics, or optical fibre sensors—at the structural level, for both a time response acquisition and strain sensing [15].

Cameras are used to visually identify the faults by acquiring pictures and/or extracting the relevant information, such as displacement measurements. A vision-based method for in-plane displacement acquisition of cantilever beams was applied in [16], while Ye et al. [17] combined a vision-based system with digital image processing and proposed a hybrid approach using a multipoint pattern-matching algorithm. Although the vision systems generally prove to capture good results when compared to other types of in situ sensors, it is found that illumination has a critical effect on the overall performance. Vision in space faces even more challenges in respect to lighting conditions, exposition, and the accessibility of partially hidden areas. According to the orbit selected, there is the chance of detectors being saturated or blinded by direct sun light, as well as interference and a poorer performance due to the noisy environment for the light background.

As this paper focuses on identifying local failures that could be then difficultly observed via cameras [18], the focus is given to the possibility of implementing a network of distributed sensors. In particular, accelerometers are selected as the sensing technology to acquire structural nodal acceleration time histories, as they are space qualified, easily installable, and have a low cost/mass impact, as demonstrated by the Honeywell, InnaLabs, and PCB catalogues [19]. Additionally, the presence of a distributed set of sensors, e.g., accelerometers and piezoelectrics, is of particular interest as being crucial to pave the road to another key technology, such as active structural control for spacecraft [20,21,22], especially in terms of failure-tolerant strategies.

Over the past decades, several techniques have been studied to perform a diagnosis and failure analysis for space systems [23,24]. Such strategies are still mostly based on traditional methods, such as lamb wave-based approaches [25], used to detect cracks or flaws which perturb the waves propagation, or a comparison between healthy and damaged structural parameters, as in dynamic strain measurements [26] and optimisation methods for model updates [27] or Transmissibility Functions (TF) [28]. Another class of techniques successfully assessed and tested in a real-time SHM is based on the eigenvalue perturbation theory [29,30], including both first- and higher-order perturbation methods [31]. Recent advancements in singular spectral analysis [32,33] proved that not only can this approach allow to detect degradation patterns and model features in real time (even by using a single sensor output), but it can also relevantly filter out the noise component [34], i.e., without any external filters. Moreover, data-driven approaches can be used in space applications focusing on in-orbit sensed acceleration profiles. The research in this category traditionally adopts modern signal processing methods and tries to catch a sudden change in signals caused by the occurrence of damage [35,36]. Recently, methodologies based on data analysis and information extraction, in the broad field of machine learning, are being increasingly used to address damage/failure identification problems to achieve a wider range of applicability [37,38]. In order to overcome the limitations associated to traditional neural networks solutions [39], such as real-world noise, more complex deep learning (DL) models and techniques, with higher generalisation capabilities, have been introduced as data extractors, classifiers, and predictors [40,41,42]. Such models can include also recurrent neural networks (RNN) [43] to efficiently obtain the information from time-series data. In fact, a state-of-the-art recurrent DL network, known as the Long Short-Term Memory NN (LSTM-NN), was applied to failure identification for an LSS by the authors [9] with promising results.

The purpose of this study is then to expand and test the limits of the approach proposed in the authors’ previous research [9]. Indeed, the present study focuses on a much more complex study case from the structural point of view, different from both the geometrical and dynamic point of view, and on the possibility to detect diverse failure types (not only in the antenna attachment area with the satellite but also on the supporting structure) that are quite difficult to detect due to a neglectable impact on the satellite dynamics. Moreover, the concept of “damage” is now extended to include not only complete failures of structural elements but also partial damages that are notoriously more difficult to classify. Indeed, one of the main objectives of this paper is to propose a more generalisable deep neural network (DNN) system for the SHM and to investigate the architecture sensitivity to different damages both in terms of the locations and structural type. In this research, a data acquisition process is carried out based on attitude manoeuvres simulations, the sensor readings are correlated to the damage presence and location, and the processing is performed by using Long Short-Term Memory neural networks (LSTM-NN) layers combined with non-linear activation layers and fully connected blocks. The resultant deep learning model is then trained to solve the sequence-to-label classification issue of structural damage detection.

The paper is divided as follows. Firstly, the fully coupled 3D mathematical model of a flexible spacecraft is implemented in a simulator for carrying out a wide set of in-orbit attitude manoeuvres (Section 2). Then, the spacecraft test case model is described, including the network of distributed sensors for the SHM and the damage configurations addressed herein (Section 3). In Section 4, the implemented deep neural architecture for the damage classification is described, based on an LSTM variant. Section 5 reports the main results and analyses the performance of the trained classification network. Finally, Section 6 discusses the relevant findings while Section 7 highlights some of conclusions and provides the indications about further steps in this research effort.

## 2. Dynamics of a Flexible Spacecraft

This section deals with the general equations of a flexible spacecraft in a gravitational field. The main steps for the description of the motion of a flexible body are:

- The definition of kinematic parameters for flexible structures;
- The definition of the functionals: kinetic, elastic, and gravitational;
- The definition of the Lagrangian;
- The writing of the equilibrium equations through the Hamilton’s principle.

To write the above-mentioned quantities, the position of a generic point *P* belonging to the spacecraft in the inertial reference frame $R_{i}$ can be expressed as the superposition of a rigid motion plus a combination of structural modes:

(1) $$x_{P}^{I}=x_{O}^{I}+P_{B/I}\left(\xi +\sum_{k=1}^{N}A_{k}\left(t\right)\phi_{k}\left(\xi \right)\right)$$

where $x_{O}^{I}$ is the position of a reference point *O* of the body in $R_{i}$, ξ is the rigid component of the position vector of point *P* with respect to the reference point *O* in the body reference frame $R_{b}$ while its flexible component is expressed via the classical modal decomposition through *N* modal amplitudes $A_{k}$ and their relevant mode shapes $\phi_{k}\left(\xi \right)$. Finally, $P_{B/I}=P_{I/B}^{T}$ is the rotation matrix that transforms vectors from $R_{b}$ to $R_{i}$.

Using Hamilton’s principle [44], the equilibrium equations of a flexible spacecraft can be obtained by evaluating the functionals associated to: the kinetic energy, the elastic potential of the system, the gravitational field, and the control actions (if any). Only the final equations of motion, Equations (2)–(4), are reported in this work for brevity’s sake while the methods leading to their derivation are covered in [44,45,46]. Hence, the governing translational, rotational, and modal base flexible equations of a flexible body under gravitational force, respectively, can be written as:

(2) $$m{\ddot{x}}_{O}^{I}+\omega \times \left(\omega \times \tilde{p}\right)+2\omega \times \sum_{k=1}^{N}\Lambda_{k}{\dot{A}}_{k}+\dot{\omega }\times \tilde{p}+\sum_{k=1}^{N}\Lambda_{k}{\ddot{A}}_{k}=f_{g}$$

(3) $$\tilde{p}\times {\ddot{x}}_{O}^{I}+\tilde{J}\dot{\omega }+\sum_{k=1}^{N}\left({\tilde{J}}_{1}^{k}{\dot{A}}_{k}\right)\omega +\sum_{k=1}^{N}\sum_{t=1}^{N}\Xi^{kt}{\dot{A}}_{k}{\dot{A}}_{t}+\sum_{k=1}^{N}{\tilde{\Gamma }}_{k}{\ddot{A}}_{k}+\omega \times \tilde{J}\omega +\omega \times \sum_{k=1}^{N}{\tilde{\Gamma }}_{k}{\dot{A}}_{k}=C_{g}+C_{c}$$

(4) $$\Lambda_{k}^{T}{\ddot{x}}_{O}^{I}+{\tilde{\Gamma }}_{k}^{T}\dot{\omega }+{\ddot{A}}_{k}+\omega_{k}^{2}\;A_{k}+2\zeta_{k}\omega_{k}{\dot{A}}_{k}-\frac{1}{2}\omega^{T}{\tilde{J}}^{k}\omega +2\omega^{T}\sum_{t=1}^{N}\Xi^{kt}{\dot{A}}_{t}={\tilde{f}}_{g,k}\;\;\;\;k=1,\ldots ,N$$

where the symbol × indicates the cross product operation between two vectors; ω is the angular velocity of the spacecraft defined with respect to $R_{i}$; *m*, $\tilde{p}$, and $\tilde{J}$, respectively, are the total mass of the spacecraft, the static moment, and its inertia tensor. The translational and rotational modal participation factors, accounting for the most significant contribution to the coupling between rigid and flexible dynamics, are, respectively, expressed as $\Lambda_{k}$ and ${\tilde{\Gamma }}_{k}$. $\omega_{k}$ and $\zeta_{k}$ are the natural frequency and damping factor associated to the *k*-th mode of vibration. Further parameters, such as $\Xi^{kt}$ and ${\tilde{J}}_{1}^{k}$ (first variation in the inertia tensor due to the flexibility), appear in higher-order terms in Equations (3) and (4).

Moreover, the gravitational forces $f_{g}$ (in the inertial reference frame $R_{i}$), the gravity gradient torques $C_{g}$ (in the body reference frame $R_{b}$), the generalised gravitational forces projected on the *k*-th elastic mode ${\tilde{f}}_{g,k}$ are reported as follows:

(5) $$f_{g}=-m\mu_{⊕}\frac{\hat{s}}{{\left|x_{O}^{I}\right|}^{2}}\;\;\mathrm{with}\;\hat{s}=\frac{x_{O}^{I}}{\left|x_{O}^{I}\right|}$$

(6) $$C_{g}=-\frac{\mu_{⊕}}{{\left|x_{O}^{I}\right|}^{2}}\left(\tilde{p}\times \hat{s}\right)+\frac{3\mu_{⊕}}{{\left|x_{O}^{I}\right|}^{3}}\left(\hat{s}\times J\hat{s}\right)$$

(7) $${\tilde{f}}_{g,k}=-\frac{\mu_{⊕}}{{\left|x_{O}^{I}\right|}^{2}}\left({\hat{s}}^{T}\Lambda_{k}\right)$$

being $\hat{s}$ the unitary vector of the position of the origin of $R_{b}$ in the inertial frame $R_{i}$ and $\mu_{⊕}$ the gravitational constant of Earth. Finally, $C_{c}$ is the vector of the applied control torques.

For more details about the symbols reported in the above equations, please refer to [44,47].

## 3. Spacecraft Model and Dataset Generation

To test the damage classification DL architecture, the case of a spacecraft equipped with an LMRM is considered. Such a model, representative of a realistic Earth Observation (EO) satellite, is composed of a central platform of prismatic shape, two solar arrays (3 × 8 m), and a large mesh reflector (diameter ∼ 12 m).

**Figure 1:**
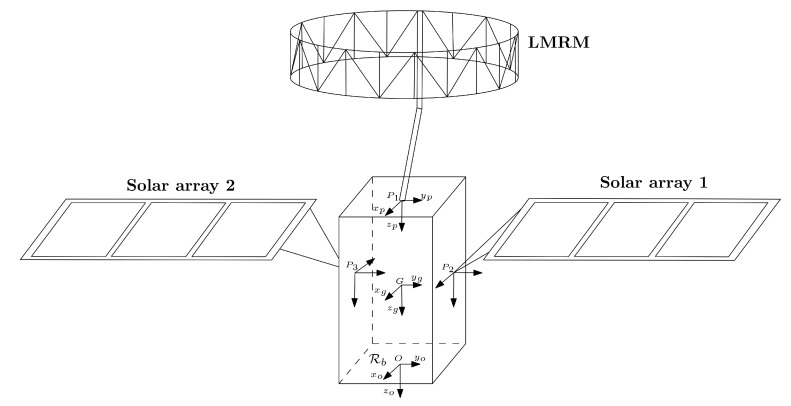
Schematic of the spacecraft model.

In detail, the LMRM is attached to a central platform—considered to be rigid with respect to the flexible structure—in correspondence to an attachment point $P_{1}$ defined in the S/C reference frame, whose origin *O* is located at the centre of the launch vehicle payload adapter, while the two large solar panels are connected to the bus at points $P_{2}$ and $P_{3}$ (see Figure 1). Each array is reinforced via a supporting structure in Carbon-Fibre-Reinforced Polymers (CFRP) and a Yoke is added to reproduce the attachment of the panel to the platform. Because the central hub is assumed rigid, each flexible substructure is directly assembled in MSC Nastran by connecting them via rigid body element connections to a single node coinciding with the point *O*. Then, the relevant data concerning the inertial properties, modal participation factors, and the natural frequencies of the flexible structure computed with respect to *O* are imported in the Matlab environment, implementing the dynamics of the flexible spacecraft described in Section 2. The inertial properties of the platform, without considering the appendages, are listed in Table 1.

### 3.1. Large Mesh Reflector Structural Model

This section briefly introduces a representative case of a realistic LMRM, which was designed using MSC Nastran FEM tool based on information available in the literature [48]. The LMRM is here composed of a circular truss backbone structure, a mesh reflector, support cables, and a deployable boom. In detail, the truss structure counts 30 unit bays, while the parabolic surface (with an areal density of about 0.3 kg/m^2^) is sustained by a net of rod elements representing the reflector cable mesh. Concerning the LMRM, the horizontal and vertical truss tubes are designed to be 2.5 m long (external radius 0.021 m, internal radius 0.02 m), while the diagonal ones 2.81 m (external radius 0.01 m, internal radius 0.009 m). The selected material for the truss is Carbon-Fibre-Reinforced Polymer (CFRP), with density equal to 1550 kg/m^3^ and Young’s modulus 125 GPa. The cable mesh is represented by rod elements with area equal to 1 mm^2^, in CFRP material. The reflector’s main structural properties are listed in Table 2. An 8 m long, 20 kg, extendable boom is also modelled to complete the structural model of the LMRM.

**Figure 2:**
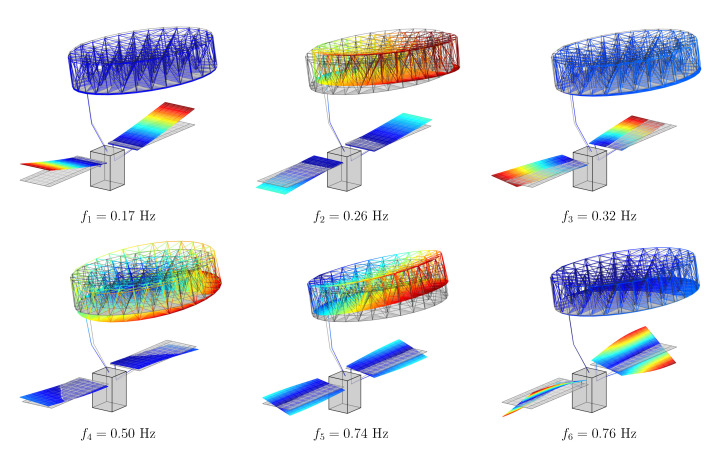
Mode shapes and natural frequency of the assembled spacecraft.

Generally, LMRM-deployed structures show three different constrained modes, namely yaw, pitch, and roll modes. They correspond to torsion and bending with respect to the main coordinated axes. When mounted on a satellite hosting other flexible appendages, the flexible modes of the payload interact with the structural dynamics of the additional substructures. In particular, the presented study case is composed of a central bus, equipped with both symmetric solar panels and the LMRM. The set of modes of the assembled spacecraft is illustrated in Figure 2, along with an overview of the complete system. It can be noticed how the system modal behaviour is characterised by both symmetric/asymmetric bending and torsion modes of the solar panels, interacting with the three traditional modes of the LMRM payload.

### 3.2. Training Set Generation and Processing

**Figure 3:**
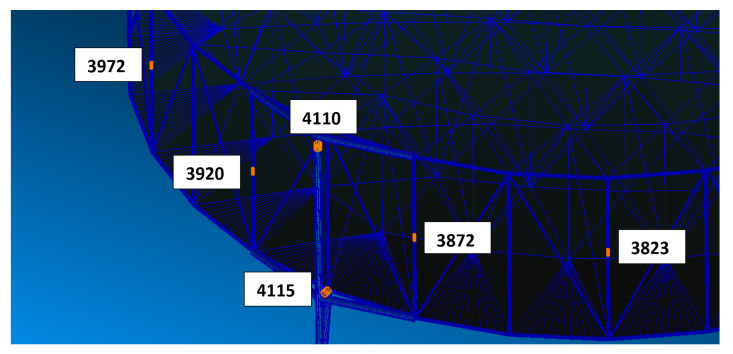
Possible damage location on the reflector structure (labels are used to identify the element in which the failure can occur).

With respect to the location of damage on the structure of the spacecraft, failures are expected to lead most likely to major consequences in the attachment element of the mesh reflector to the satellite. In the following, as reported in Figure 3, six possible structural elements of the reflector are supposed to be susceptible to a failure. In particular, failures are placed in those areas where issues may occur, such as connection areas, where, for instance, hinges or similar components are located due to the structure deployment strategy. Moreover, they are considered at both the attachment area of the antenna with the satellite and on the supporting truss to test the failure/damage of elements with completely different impact on the overall satellite dynamics. Furthermore, for each element, two damage scenarios are considered: the first one consists of the complete failure of the structural element (“Broken element” in Table 3) while the second models a partial damage of the element itself (“Damaged element” in Table 3). To mathematically reproduce these behaviours, the “Broken elements”, associated to the fully damaged location of the structure, are erased from the finite element model (in other words, the structural stiffness is considered null, and the order of the stiffness matrix is changed), while the partial structural failure, on “Damaged elements”, is introduced into the structure considering a 50% reduction in the element’s Young’s modulus, which is representative of the mechanical properties of the material of the damaged element. Table 3 also reports the associated label to each elements and damage type used to train the classification DL network.

**Figure 4:**
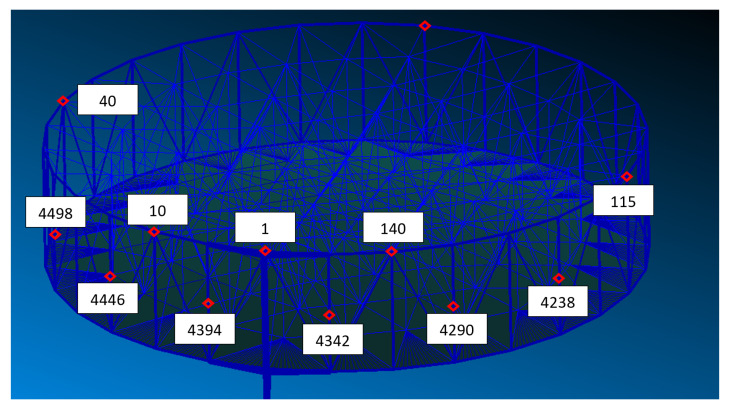
Network of distributed sensors (labels are used to identify the node on which the sensor is placed).

The behaviour of the LMRM during its operational life is observed by a network of sensors that has been designed in order to measure the time response of the structure in some critical points, namely Nodes of Interest (NOIs), of the FEM (see Figure 4). In detail, a set of 12 tri-axial accelerometers is considered, with the majority of them localised in proximity to critical areas, such as the connection of the reflection with the boom.

**Figure 5:**
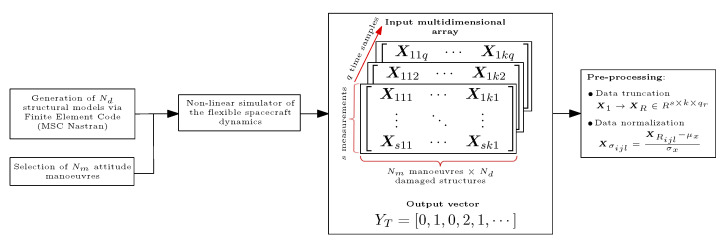
Dataset generation and processing prior to network training.

Once the damaged structural sub-models, deriving from the original undamaged one, and the network of sensors are defined, the dataset generation for the training of the DNN can be set up. This process, summarised in Figure 5, consists of the following steps:

- The set $N_{d}$ structural models extrapolated from the finite element suite (MSC Nastran), as previously described, are imported in Matlab to perform further analysis. At this point, the non-linear simulator of the flexible spacecraft (built on the basis of the equations reported in Section 2) is used to carry out $N_{m}$ attitude manoeuvres and produce the measurements from the sensors network. A quaternion-based PD controller [49,50] is implemented to produce the desired control torque $C_{c}$:
  (8) $$C_{c}=-K_{p}{\bar{q}}_{e}^{I}\mathrm{sign}\left(q_{0}\right)-K_{d}\omega$$
  with $K_{p}$ and $K_{d}$ as proportional and derivative gain matrices, respectively, ${\bar{q}}_{e}^{I}$ the error quaternion [50], $q_{0}$ scalar component of the quaternion, and ω the angular rate of the spacecraft. The dataset of possible manoeuvres is built by varying properly both the desired final attitude angles of the manoeuvre (considering also one- and two-axis manoeuvres to improve the variation in the data collection) and the gains of the controller.
- *s* quantities of interest (i.e., s=36 because 12 tri-axial accelerometers are mounted on the structure) are then collected by the sensors network with a sampling frequency of 10 Hz. At this stage, the raw data, extrapolated from the simulator, consists of a multidimensional array $X_{1}\in R^{s\times k\times q}$ with $k=N_{d}\cdot N_{m}$ and *q* the number of time samples. Moreover, a Gaussian noise equal to the 2% of measured values is considered when acquiring the data from the accelerometers to simulate a realistic condition.
- The raw multidimensional array $X_{1}$ is not directly fed to the network for the training, but it passes through two intermediate steps of pre-processing. Indeed, data-driven classification algorithms need a relevant amount of training data, which require to be properly processed to avoid introducing biases and ill-conditioning in the results, while being used in a computationally and time-expensive process (in particular for recurrent neural networks). In this paper, firstly, each time sequence is truncated ($X_{1}\to X_{R}\in R^{s\times k\times q_{r}}$) in order to preserve only parts of the signals where relevant dynamical content is detected. In the current study, the sequences are truncated to 25s for a total of 251 samples. Such a reduction is supported by the fact that responses below a certain threshold do not improve classification accuracy during network training or operation. Conversely, it was also observed in [9] that feeding the full-time sequence to the network rather jeopardises the performance of the training process (and subsequently also the performance of damage identification in real-time condition). The second step consists of normalising the measurements so as to ensure proper dynamic range of the variables in the learning space of the NN model. Therefore, in this study, each time sequence is normalised according to their mean and standard deviation as reported here:
  (9) $$X_{\sigma_{ijl}}=\frac{X_{R_{ijl}}-\mu_{x}}{\sigma_{x}}\;\;\mathrm{with}\;\;\begin{array}{cc} i & =1,\ldots ,s\\ j & =1,\ldots ,k\\ l & =1,\ldots ,q_{r} \end{array}$$
  where $\mu_{x}$ and $\sigma_{x}$ are, respectively, the mean value and standard deviation of the measured time history $\left[X_{R_{ij1}}\;X_{R_{ij2}}\;\cdots \;X_{R_{{ijq}_{r}}}\right].$

**Figure 6:**
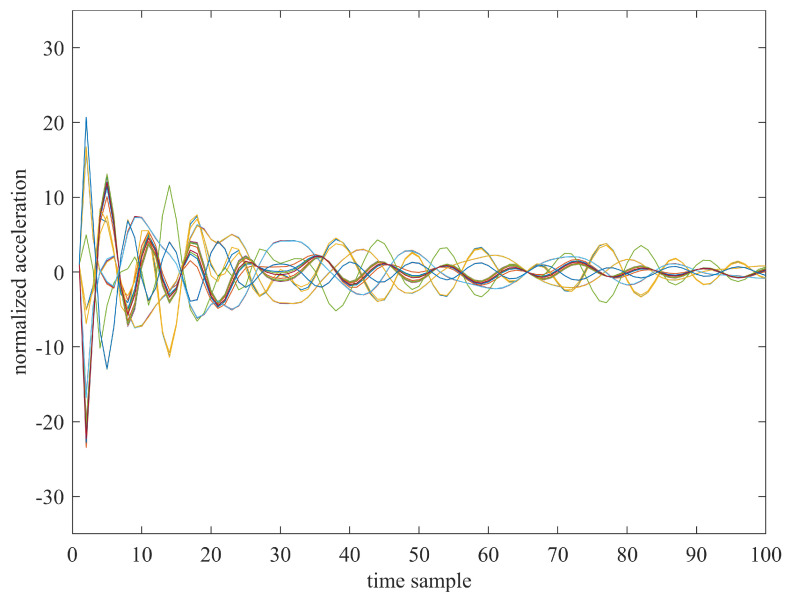
Time−series behaviour (first 100 time samples) of the 36-sensor data relevant to one of the 231 observations of the dataset with the undamaged system (class label “0”).

For the sake of illustration, we show, in Figure 6, the behaviour of the truncated and normalised 36-sensor time series associated with one observation having class label “0” (undamaged system). The picture aims at pointing out that the most relevant dynamic content of the acceleration time sequences is detected in the first-time instants of the manoeuvre. Indeed, after the rotation of the satellite has occurred, the residual naturally damped oscillations fade out and the system returns to a stationary condition while the measurements go to zero. Therefore, the time histories have been cut to avoid including in the dataset the components (equal to zero or under a certain threshold) which would contribute to flatten the dataset. The final dataset used for the training consists of an input multidimensional array $X_{R}$ of size 36 × 3003 × 251 while the output vector contains the labels of the failures reported in Table 3 and it has dimensions equal to 3003 × 1, where each simulation is associated with the label of the corresponding failure.

## 4. Bidirectional Long-Short Term Memory Network

In recent times, deep recurrent neural networks have been the staple for time-series classification problems. In particular, because most of the real-world use cases revolve around sequences that are in the hundreds or thousands of observations, it was deemed necessary to utilise learning models that are able to connect information found far in the past to the present task. In this regard, LSTM networks are a well-known solution and have been applied to a multitude of problems. While the LSTM model is indeed reliable, there are some cases in which a more robust information extraction is needed, such as the classification of long sequences. To this end, bidirectional Long Short-Term Memory (Bi-LSTM) networks have been introduced and applied to many practical contexts [51].

**Figure 7:**
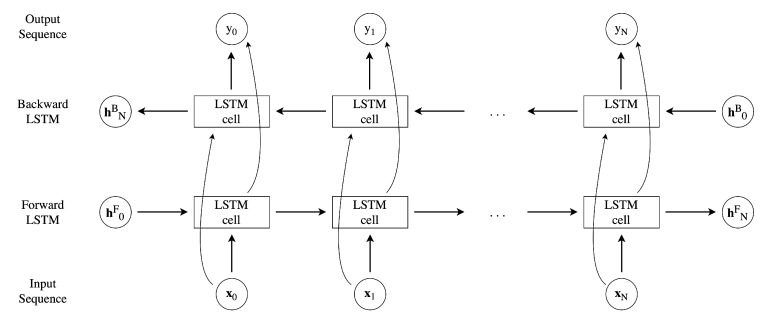
Unrolled network structure of a bidirectional LSTM.

Unlike the classic, unidirectional LSTM model, the Bi-LSTM applies a first LSTM on the input sequence in the given order, then it reverses the sequence and feeds the second LSTM. It was proved [52] that the Bi-LSTM architecture works better in a multivariate time-series analysis and classification, which is the application studied in this work. A detailed scheme of the workings of the Bi-LSTM model is reported in Figure 7. The input size is equal to *N* and the hidden state ${h^{i}}_{j}$ refers to the *j*-th time step of the *i*-th (backward or forward) LSTM.

It is important to underline that the Bi-LSTM is trained twice, once for both directions (forward and backward). The bidirectional application of this more complex LSTM cell structure is performed to robustly save information from both past and future samples, improving the accuracy of the model [53].

In the present work, we employ a multivariate deep classification model which we called deep Bi-LSTM (DBLSTM); it is composed of an input layer (in the form of a sequence) and two stacked Bi-LSTM layers, interspersed with a dropout layer, a dense layer, a Softmax layer, and a classification layer. A qualitative trade-off was carried out between the different network architectures to find the best compromise between the computational costs and accuracy. Therefore, the network proposed in this paper was selected as the simplest stack that could be applied to this configuration.

**Figure 8:**
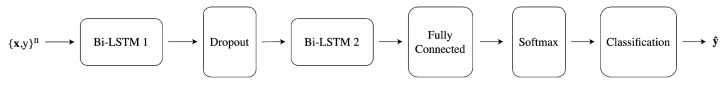
Network configuration of the proposed DBLSTM.

The sequence input layer creates the proper input sequence to feed the network. The two Bi-LSTM layers are the ones charged with learning the temporal dependencies among the data; two stacked layers are preferred over a single layer to enhance the predictive power of the model [54]. A dropout layer is inserted between the Bi-LSTM layers as a regularisation factor to avoid overfitting. A fully connected (FC) layer is stacked on the last Bi-LSTM unit to map the output of the Bi-LSTM layer to a desired output size. The Softmax layer turns the output of the FC layer into probability values summing to one so that the final classification layer is able to perform the binary classification. The architecture of the network is visualised in Figure 8.

The output of the model is a binary vector $\hat{y}$, whose dimension is equal to *n* and which contains the predicted outcome.

In the DBLSTM scheme, the number of hidden units in the Bi-LSTMs is denoted as $N_{h}^{\left(1\right)}$ for the first layer and $N_{h}^{\left(2\right)}$ for the second layer; these are the recurrently connected blocks (i.e., the computational units) and their number should be optimised based on the application and data purposes.

In fact, based on the dataset defined earlier, it is possible to empirically set the hyperparameters for the DBLSTM network structure; the first Bi-LSTM layer has 36 features and two or three classes in the FC and Softmax layers, depending on the specific problem, as explained in Section 5.

## 5. Results

The present study investigates the sensitivity and accuracy of the structural damage recognition by using an ensemble of sensors distributed across the structure to be monitored. We dealt with this problem by using a suited DNN architecture for the multivariate time-series classification, namely the one introduced in Section 4, and the dataset obtained by the measures through the simulations on the spacecraft model defined in Section 3.

We carried out three different types of analysis; in every problem, all the sensor data are used to build a 36-element vector associated with each time sample of the multivariate time series that feed the input of the adopted DNN:

- First, six binary classification problems were considered, one for each structural element reported in Table 3. Two classes only are selected among the observations: 231 time series having the class label “0” (undamaged system) and 462 times series having a unique class label associated with that element, either broken or damaged (i.e., a unique class named “1-2” for “Elm 4115”, a unique class named “3-4” for “Elm 4110”, and so on). This meets the normal logic of damage detection to firstly determine whether damage has occurred or not and then to determine the degree of the damage.
- Successively, twelve binary classification problems were considered, two of them for each structural element reported in Table 3. Two classes only are selected among the observations: 231 time series having the class label “0” (undamaged system) and 231 times series having one class label (between “1” and “12”), which is associated with a specific failure of the considered structural element. Here, the rationale is to find whether, by analysing the data coming from all the sensors, is it possible to recognise one specific failure at a time, either a broken or damaged element, with respect to an undamaged condition.
- The third analysis pertains to six three-class classification problems, one for each structural element reported in Table 3. Three classes are considered in this case: 231 time series having the class label “0” (undamaged system); 231 times series having an odd class label (between “1” and “11”), which is associated with the broken failure of the considered structural element; and 231 times series having the even class label (between “2” and “12”) associated with the damaged failure of the same element, which is the previous odd class label increased by one. In this situation, we want to study the capability of the proposed multivariate deep learning approach to identify both a broken and a same damaged element with respect to the undamaged condition.

The optimal model performance and selection of the considered deep neural architecture were obtained by means of a nested cross-validation procedure. Namely, an outer 10-fold cross-validation was carried out for a model performance evaluation. In each of these folds, using the training data only, a grid search procedure was applied to obtain the best setting of the hyperparameters for an optimal model selection. For each hyperparameters’ setting (i.e., for each point of the grid to be searched), an inner 3-fold cross-validation was carried out for obtaining different partitions into training and validation sets in order to actually train the neural network with the ADAM algorithm [55].

Because of the randomness in any k-fold partitioning and in neural network parameters’ initialisation, both the outer and inner cross-validations were repeated for 10 and 5 runs, respectively, using a different seed for the random number generation. Accordingly, the statistical average of the classification accuracy on the validation set, over the different inner folds and runs, was used for the best hyperparameters’ selection during the grid search; the statistical average of the classification accuracy on the test set, over the different outer folds and runs, was used to evaluate the final model performance, and it is reported in the following tables.

The hyperparameters optimised in each grid search were the number of units $N_{h}^{\left(1\right)}$ and $N_{h}^{\left(2\right)}$ in the first and second Bi-LSTM layer of the adopted network, respectively. The possible values for both of them were searched in the range from 5 to 50 with a step of 5 units. We note that the optimal values of the hyperparameters, which are obtained at the end of every grid search procedure, generally differ among the several outer folds and hence only a statistical average of the optimal setting can be reported in the following tables. The other main hyperparameters set in advance were a dropout rate of 0.1; 150 maximum epochs; five iterations per epoch (which affects the mini batch size according to the actual training size); an ADAM learning rate of 1.0 (0.75% drop factor every 10 epochs); a regularisation factor of 0.0001; a gradient decay factor of 0.9; and the validation patience of 50 epochs before early stopping.

All the experiments were performed using Matlab^®^ R2022a on a machine provided with an AMD Ryzen Threadripper^™^ 3970X CPU (32 cores at 4.5 GHz), 128 GB of DDR4 RAM (at 3.2 GHz), and an NVIDIA^®^ RTX A6000 GPU (10752 cores at 1.8 GHz) with 48 GB of GDDR6 RAM (bus width 384 bits at 16 GB/s).

The numerical results for the two-class recognition problems described before, both the six and twelve arrangements, are reported in Table 4. As explained, the accuracy is the average on the several test sets while the optimal values of the Bi-LSTM slightly differ among the different optimisation tests. It is evident that the structural element “Elm 4110” can be well recognised both in the case of explicit breaking (class “3”) or damage (class “4”); also, the prior discrimination as to whether general damage occurred (class “3-4”) or not is obtained with a maximum accuracy close to 100%. The element “Elm 4115” is well classified in the case of explicit breaking only (class “1”), while the prior discrimination of general damage (class “1-2”) is less accurate but good enough around 75%.

The other failures are recognised at a poor rate around 50% in the case of either broken or damage recognition. In these cases, the prior analysis as to whether general damage has occurred or not seems more accurate around 66.7%, but this is due only to the unbalanced datasets used in such situations, as the undamaged time series are 231 while the ones associated with general damage (either the “broken” or “damaged” class) are doubled to 462. Nonetheless, a discussion in this regard is reported in the next section. We also note that the optimal values of the Bi-LSTM units are always considerably smaller than 50, which was the largest value considered during the grid search; thus, the overfitting of the tested networks was correctly prevented.

**Figure 9:**
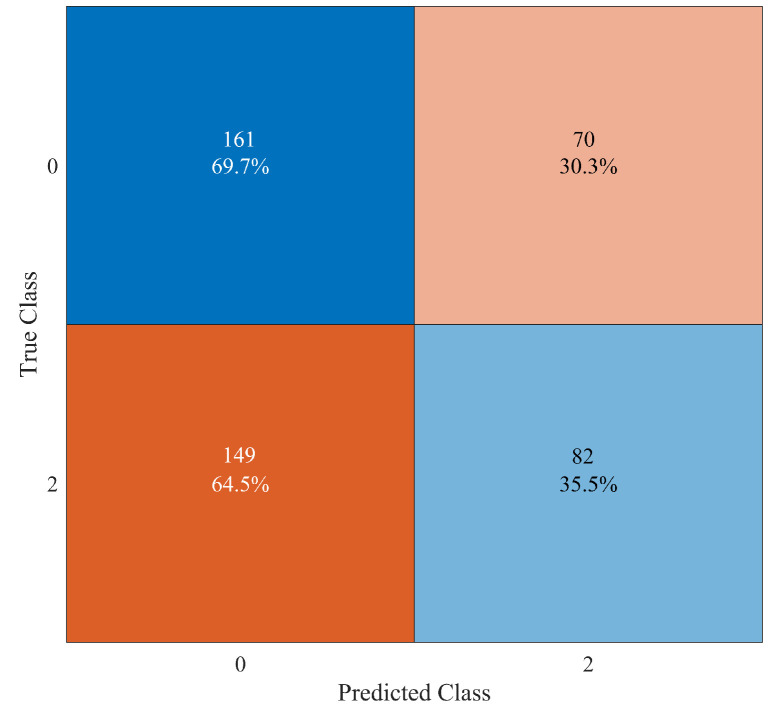
Confusion matrix for the binary classification between undamaged system (class “0”) and “Elm 4115” damaged (class “2”).

**Figure 10:**
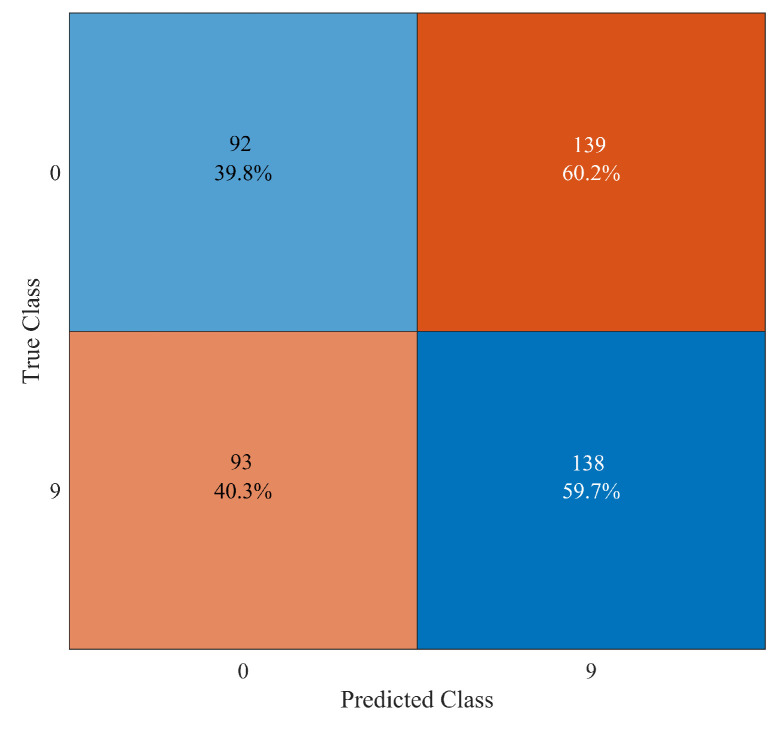
Confusion matrix for the binary classification between undamaged system (class “0”) and “Elm 3823” broken (class “9”).

It is interesting to show which kind of mistakes are made in wrong situations. For instance, in Figure 9, the confusion matrix in the case of “Elm 4115” damaged (class “2”) is reported. In this case, the number of undamaged observations that are misclassified as “Elm 4115” damaged (i.e., false positive) is 70, which is less than the 149 observations of “Elm 4115” damaged that are misclassified as undamaged (i.e., false negative). Conversely, as shown in Figure 10 in the case of “Elm 3823” broken (class “9”), the number of false positive observations is 139, which is larger than the 93 false negative observations. So, no general rules can be proved to exist about the majority of false positive or false negative observations.

The numerical results for the three-class recognition problem are reported in Table 5. It is confirmed that, also in this case, element “Elm 4110” is well-classified in both situations; in fact, the accuracy is close to 100%. Similarly, “Elm 4115” is well-classified only in part of the situations, therefore achieving an accuracy close to 72%. The failures of the other elements are poorly recognised, staying always around 33% (i.e., 1 out of 3). Moreover, in this case, the optimal values of the Bi-LSTM units are far from the largest possible value of 50, thus showing that the optimal networks are not overfitted.

**Figure 11:**
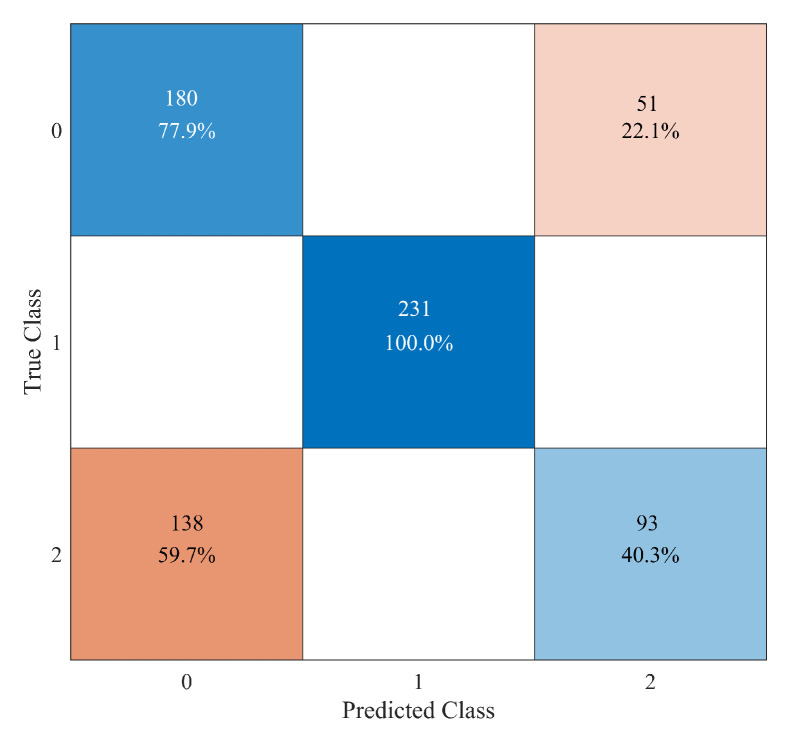
Confusion matrix for the 3-class recognition problem regarding the undamaged system (class “0”) and the failures of element “Elm 4115” (class “1” and “2”).

**Figure 12:**
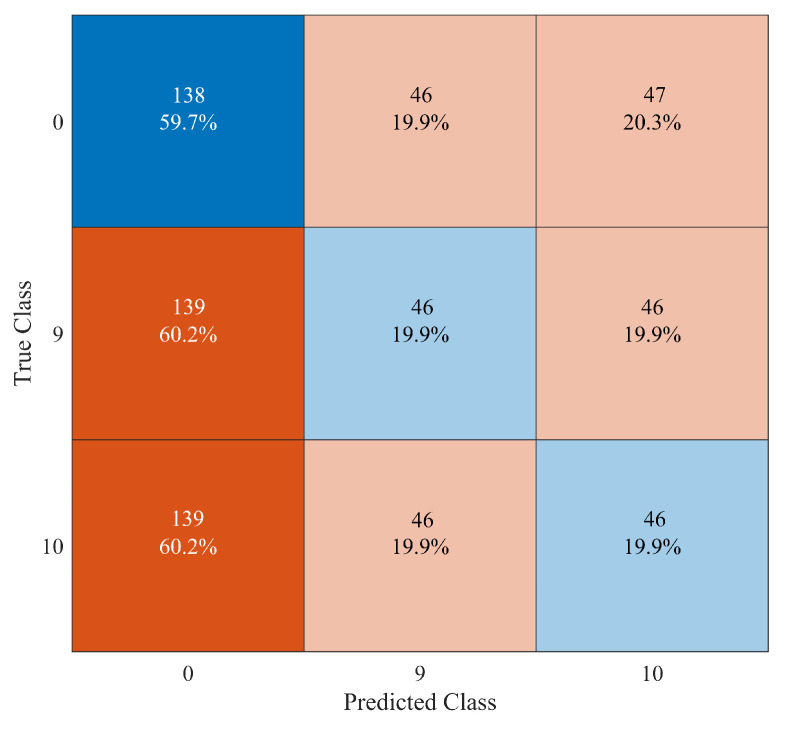
Confusion matrix for the 3-class recognition problem regarding the undamaged system (class “0”) and the failures of element “Elm 3823” (class “9” and “10”).

By looking at the confusion matrix shown in Figure 11, we see that the broken situations of “Elm 4115” (class “1”) are always perfectly classified. However, there are 51 false positive classifications of the undamaged system as “Elm 4115” damaged (class “2”) and 138 false negative classifications of “Elm 4115” damaged as an undamaged structure. By the way, these numbers are similar to the case of the binary classification reported in Figure 9, therefore showing a peculiarity on some time series related to this structural element when damaged. A totally different situation pertains to other elements with a poor classification accuracy, for instance, as illustrated in Figure 12 for element “Elm 3823”. In this case, there is an even distribution of false positive classifications (i.e., 46/47 cases) between class “9” and “10”, as well as an equal distribution of 139 false negative recognitions of an undamaged system instead of a broken or damaged element.

## 6. Discussion

The obtained outcome is comprehensively interpreted and discussed in Table 6. The analysed sensing architecture proved to be optimal to classify the damages for the elements near the antenna attachment area with the central satellite. It can be noticed, however, that the presented results clearly depend on the physical location of the damage with respect to the sensors network and, simultaneously, on the physical properties of the system itself. This opens the possibility to future investigations on the optimal positioning of sensing devices with respect to the most sensitive structural elements based on machine learning approaches, with particular regards to the problem of investigating and differentiating similar data originating in different attitude manoeuvres.

## 7. Conclusions

This paper investigated the problem of detecting structural breaks and partial damages for a complex system, such as a large space antenna, while assessing the SHM framework/sensors sensitivity to local damages with regard to their geometric location and impact on the system dynamics. The adopted data-driven SHM approach was based on a state-of-the-art deep recurrent neural network (Bi-LSTM) with a tailored structure for damage classification. The proposed DL architecture was trained with data produced by using a 3D simulator of the attitude/flexible dynamics of a spacecraft equipped with structural appendages, including not only a mesh antenna but also a pair of symmetric solar panels. The obtained results proved the SHM system was able to accurately identify breaks in the antenna attachment area with the hosting spacecraft while showing a lower performance in the case of distributed failures in the backbone antenna supporting elements. Therefore, for the considered study case, a minimal set of two sensors is deemed sufficient to detect those failures which could produce detectable changes in the system dynamics.

Future research will deepen the obtained results by using the DL approach to investigate optimal sensors placement configurations for different classes of large space structures. Moreover, an improvement in the presented outcome will be explored by implementing upgraded DL strategies, such as methods augmented/coupled with a certain degree of knowledge of the physical system. This would aim at discriminating among the specific types of failures and/or similar acquired data and to knowingly address areas judged to have a minimal impact on the overall system dynamics. A possible approach could be the use of physics-informed machine learning techniques to better discern the types and location of damages, thus improving the system sensitivity.

Further work might also consider adopting automatic validation/testing methods to properly tune the complexity of the proposed network (based on the type and number of implemented sensors) and to use the DL approach to assess the best sensors to detect a specific structural failure or damage.

## Figures and Tables

**Table 1:** Platform inertial properties.

Mass	Inertia Wrt CoM						CoM Wrt $R_{b}$		
(kg)	$\left(\mathrm{kg}m^{2}\right)$						(m)		
	Jxx	Jyy	Jzz	Jxy	Jxz	Jyz	X	Y	Z
1200	903.6	985.2	885.6	2	4	−7	0	0	−2

**Table 2:** Reflector properties.

Property	LMRM
Mass (kg)	Truss: 38.34
	Mesh: 4.25
	Total: 42.6
1st Mode (Hz)	Truss: 0.20
	Truss + Mesh: 0.53

**Table 3:** Damaged element ID, associated damage configuration (broken or partial failure), and associated label.

Damaged Element ID	Damage Configuration	Associated Label
-	Undamaged system	0
Elm 4115	Failure 1—Broken element	1
	Failure 2—Damaged element	2
Elm 4110	Failure 3—Broken element	3
	Failure 4—Damaged element	4
Elm 3872	Failure 5—Broken element	5
	Failure 6—Damaged element	6
Elm 3920	Failure 7—Broken element	7
	Failure 8—Damaged element	8
Elm 3823	Failure 9—Broken element	9
	Failure 10—Damaged element	10
Elm 3972	Failure 11—Broken element	11
	Failure 12—Damaged element	12

**Table 4:** Classification Results of the 2-class Structural Health Recognition Problems.

Failure	Class Labels	Accuracy (%)	Bi-LSTM Units 1	Bi-LSTM Units 2
Elm 4115 broken–damaged	0,1-2	75.8±4.9	20±10.0	11±4.2
Elm 4115 broken	0,1	100.0±0.0	30±12.2	12±2.7
Elm 4115 damaged	0,2	47.3±8.5	14±5.5	13±2.7
Elm 4110 broken–damaged	0,3-4	99.9±0.3	22±4.5	6±2.2
Elm 4110 broken	0,3	99.8±0.5	16±5.5	5±0.0
Elm 4110 damaged	0,4	100.0±0.0	22±11.0	6±2.2
Elm 3872 broken–damaged	0,5-6	66.7±0.3	16±8.9	14±5.5
Elm 3872 broken	0,5	58.8±21.3	18±11.0	16±6.5
Elm 3872 damaged	0,6	50.4±1.0	32±17.9	10±5.0
Elm 3920 broken–damaged	0,7-8	66.7±0.3	20±17.3	10±7.1
Elm 3920 broken	0,7	54.3±7.5	26±21.9	15±6.1
Elm 3920 damaged	0,8	50.0±0.4	26±16.7	8±4.5
Elm 3823 broken–damaged	0,9-10	67.1±1.1	28±20.5	10±6.1
Elm 3823 broken	0,9	49.8±0.3	18±8.4	11±8.2
Elm 3823 damaged	0,10	50.0±0.4	16±8.9	6±2.2
Elm 3972 broken–damaged	0,11-12	66.7±0.3	18±7.9	8±4.5
Elm 3972 broken	0,11	50.0±0.4	32±17.9	6±2.2
Elm 3972 damaged	0,12	49.8±0.3	30±14.1	9±6.5

**Table 5:** Classification Results of the 3-class Structural Health Recognition Problem.

Failure	Class Labels	Accuracy (%)	Bi-LSTM Units 1	Bi-LSTM Units 2
Elm 4115 broken, damaged	0,1,2	72.7±3.0	32±11.0	11±4.2
Elm 4110 broken, damaged	0,3,4	99.8±0.3	18±8.4	8±2.8
Elm 3872 broken, damaged	0,5,6	33.8±0.5	20±7.1	8±4.5
Elm 3920 broken, damaged	0,7,8	34.5±6.0	22±11.0	16±6.5
Elm 3823 broken, damaged	0,9,10	33.2±0.1	22±8.4	8±6.7
Elm 3972 broken, damaged	0,11,12	33.3±0.3	32±17.9	14±6.5

**Table 6:** Results interpretation.

Structural Element	Discussion
Elm 4110	The DNN architecture proved to well detect and identify the failure, both in the break and the partial damage cases. The reason for this outcome can be interpreted as three-fold. On one hand, Elm 4110 and Elm 4115, located at the antenna attachment point with the satellite, are the ones affecting the overall system behaviour the most—yet still limitedly, even from a structural point of view—when compared to the other considered elements. Indeed, as proved by the previous research discussed in [9], failures near a substructure attachment area to a satellite can be straightforwardly identified by using LSTM-based machine learning approaches. This is true in terms of structural stiffness modifications and consequently of natural frequencies changes and different antenna nodal elastic displacements. On the other hand, local partial damage—which could be more difficult to classify due to its minor impact on the satellite attitude dynamics—can be identified with high precision mainly due to its vicinity to Sensor “1”, which is able to better “see” the local effects of the failure. Finally, the location of Elm 4110—as well as 4115—is peculiar with respect to the other elements: it is positioned in a way to not be impacted by the symmetry effects of the overall system structure and also in terms of the registered acceleration profiles (please refer to further discussions in this table for more details).
Elm 4115	The DNN architecture demonstrated to be able to well detect and classify the total break of Elm 4115. Similarly to Elm 4110, this behaviour can be assumed as due to the more relevant impact on the system dynamics than other structural elements and on its position. In detail, while a good classification performance can be observed for the three-class problem in Table 5, the partial damage cannot be identified with the same accuracy when identified with respect to the undamaged condition. This result is interpreted as related to the absence of any local sensors near the damaged element.
Elm 3972 Elm 3920 Elm 3872 Elm 3823	The DNN architecture showed not to be able to properly detect and classify both the total break and partial damage cases with respect to the undamaged condition nor in the three-class problem. This behaviour gives relevant information on the ability of detecting failures on distributed elements in relation to their position on the structure. It can be noticed, indeed, that the same classification performance can be observed for all the damaged elements, except Elm 4115 and Elm 4110. This can be explained by the fact that the structural parts are symmetrically positioned with respect to the plane, including the Z-axis, perpendicular to the Y-axis, which is indeed a symmetry plane of the satellite (please refer to Figure 1): Elm 3972 is symmetric to Elm 3823, and Elm 3920 to 3872. Likewise, Sensors “1” and “75” are the only sensors positioned in the symmetry plane, while the others are located symmetrically with respect to each other. Hence, they are inducing a comparable effect/change in the system dynamics which results in similar classification results. Indeed, according to the specific manoeuvre, they are producing either a response with the same magnitude and same sign, or the same response with opposite sign. In general, nevertheless, this proves that the local failures, both breaks and damages, have a limited impact on the system from a structural point of view. They are all indeed elements which are part of the antenna backbone supporting structure, as opposed to the attachment point. This, on the other hand, indicates that, even with local sensors near the damage/failure of the element, the damage on the supporting backbone loop does not change the properties of the system enough to be clearly identified by the DNN architecture. Because the DNN system relies purely on data, without knowledge of the physical system, which could help discriminate the area of the failure, the accuracy level is significantly worse than the one of both Elm 4115 and 4110.

## Data Availability

Not applicable.

## Abbreviations

The following abbreviations are used in this manuscript:

ANN	Artificial Neural Network
DL	Deep Learning
DNN	Deep Neural Network
ELM	Element
FEM	Finite Element Method
LMRM	Large Mesh Reflector Model
LSS	Large Space Structure
LSTM	Long Short-Term Memory
ML	Machine Learning
NN	Neural Network
RNN	Recurrent Neural Network
SHM	Structural Health Monitoring
